# HSP90 inhibition enhances cancer immunotherapy by upregulating interferon response genes

**DOI:** 10.1038/s41467-017-00449-z

**Published:** 2017-09-06

**Authors:** Rina M. Mbofung, Jodi A. McKenzie, Shruti Malu, Min Zhang, Weiyi Peng, Chengwen Liu, Isere Kuiatse, Trang Tieu, Leila Williams, Seram Devi, Emily Ashkin, Chunyu Xu, Lu Huang, Minying Zhang, Amjad H. Talukder, Satyendra C. Tripathi, Hiep Khong, Nikunj Satani, Florian L. Muller, Jason Roszik, Timothy Heffernan, James P. Allison, Gregory Lizee, Sam M. Hanash, David Proia, Rodabe Amaria, R. Eric Davis, Patrick Hwu

**Affiliations:** 1 Department of Melanoma Medical Oncology Unit 904, The University of Texas MD Anderson Cancer Center, 1515 Holcombe Boulevard, Houston, TX 77030 USA; 2 Department of Lymphoma/Myeloma Unit 903, The University of Texas MD Anderson Cancer Center, 1515 Holcombe Boulevard, Houston, TX 77030 USA; 3 Institute for Applied Cancer Sciences Unit 1956, The University of Texas MD Anderson Cancer Center, 1515 Holcombe Boulevard, Houston, TX 77030 USA; 4 Department of Clinical Cancer Prevention Unit 1013, The University of Texas MD Anderson Cancer Center, 1515 Holcombe Boulevard, Houston, TX 77030 USA; 5 Cancer Imaging Systems Unit 1907, The University of Texas MD Anderson Cancer Center, 1515 Holcombe Boulevard, Houston, TX 77030 USA; 6 Department of Immunology Unit 901, The University of Texas MD Anderson Cancer Center, 1515 Holcombe Boulevard, Houston, TX 77030 USA; 7 Synta Pharmaceuticals Inc., 45 Hartwell Avenue, Lexington, MA 02421 USA

## Abstract

T-cell-based immunotherapies are promising treatments for cancer patients. Although durable responses can be achieved in some patients, many patients fail to respond to these therapies, underscoring the need for improvement with combination therapies. From a screen of 850 bioactive compounds, we identify HSP90 inhibitors as candidates for combination with immunotherapy. We show that inhibition of HSP90 with ganetespib enhances T-cell-mediated killing of patient-derived human melanoma cells by their autologous T cells in vitro and potentiates responses to anti-CTLA4 and anti-PD1 therapy in vivo. Mechanistic studies reveal that HSP90 inhibition results in upregulation of interferon response genes, which are essential for the enhanced killing of ganetespib treated melanoma cells by T cells. Taken together, these findings provide evidence that HSP90 inhibition can potentiate T-cell-mediated anti-tumor immune responses, and rationale to explore the combination of immunotherapy and HSP90 inhibitors.

## Abstract (web summary)

Many patients fail to respond to T cell based immunotherapies. Here, the authors, through a high-throughput screening, identify HSP90 inhibitors as a class of preferred drugs for treatment combination with immunotherapy.

## Introduction

The importance of T cells in anti-tumor immunity has been established over the years, resulting in the emergence of promising T-cell-based immunotherapies such as immune checkpoint blockade. Treatment with anti-PD-1 and anti-CTLA4 immunotherapy can result in clinical responses of up to 50% in melanoma, some of which are durable^1, 2^. However, the majority of patients across different cancer types fail to respond durably to these T-cell-mediated immunotherapies. This underscores the need to further understand the factors interfering with response to immunotherapy, to better inform combination therapies.

There is increasing evidence that tumor intrinsic pathways not only promote tumorigenesis but also interfere with processes essential for an effective anti-tumor immune response, such as T-cell trafficking and T-cell-mediated killing of tumor cells. For instance, studies from our group and others have shown that oncogenic BRAF signaling in tumor cells results in the expression of immunosuppressive molecules such as VEGF in the tumor microenvironment. Inhibition of BRAF significantly augments anti-tumor immune responses through decreased expression of VEGF, increasing antigen presentation and trafficking of T cells to the tumor microenvironment^3, 4^. In addition, activation of the PI3K pathway via PTEN loss negatively affects T-cell infiltration into tumors and T-cell-mediated lysis of tumors^5^. These findings of tumor intrinsic pathways with immunosuppressive effects have informed combination therapies with immunotherapy and clinical trials are underway.

To identify additional small molecules and pathways with potential to improve responses to immunotherapy, we performed a broad screen of 850 bioactive compounds to assess their effect on killing of primary melanoma cell lines by autologous T cells. Among the results, inhibitors of the molecular chaperone heat shock protein 90 (HSP90) synergistically improved T-cell killing. We subsequently provide evidence that upregulation of interferon response genes mediates this effect, and show that the clinically relevant HSP90 inhibitor ganetespib potentiates responses to anti-CTLA4 and anti-PD-1 immunotherapy in a preclinical murine tumor model.

## Results

### HSP90 inhibition enhances T-cell killing of tumor cells

**Fig. 1:**
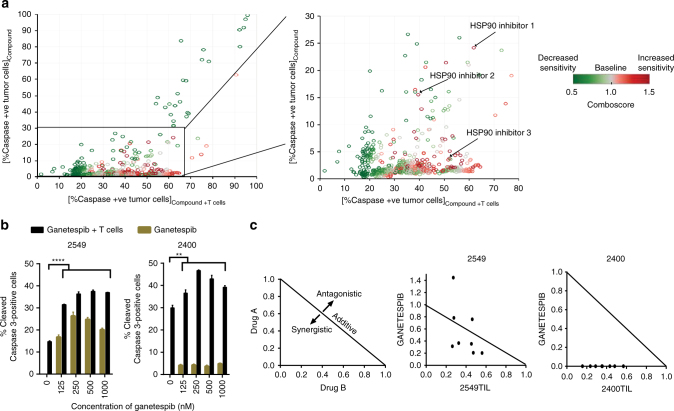
HSP90 inhibition enhances T-cell mediated killing of melanoma cells. **a** Tableau depiction of screen results from patient-derived cell line 2549. HSP90 inhibitors 1—17-DMAG, 2—BIIB021 and 3—17-AAG are highlighted. **b** Cleaved caspase 3 percentage following treatment with varying concentrations of ganetespib and autologous TILs in human melanoma cell lines 2549 and 2400. **c** Isobolograms depicting the synergism between ganetespib and T-cell killing. Points >1 indicate antagonism, points = 1 indicate additivity and points <1 indicate synergism. An unpaired two tailed Student *t*-test was performed with **P < 0.01; ****P < 0.0001. Data are a representation of at least two independent studies

To identify compounds that increase the sensitivity of human melanoma cells to T-cell mediated killing, we utilized paired patient-derived human melanoma cell lines and their autologous tumor infiltrating T cells (TILs), derived from our active adoptive cell therapy program, in a high throughput in vitro screen of 850 bioactive compounds (Supplementary Fig. 1). Two human melanoma cell lines 2549 (wild type for *BRAF*, *NRAS* and *cKIT*) and 2338 (*BRAF* V600E mutated) were treated with 1 µM of each compound for 24 h, or DMSO as a control. The treated tumor cells were then washed and incubated with autologous TILs for 3 h at a predetermined ratio, and the levels of cleaved caspase 3 assessed as a readout of apoptosis. To quantify the interactive effect of the compounds on T-cell-mediated killing, a comboscore was calculated from the percentage of TIL-induced apoptosis in tumor cells with or without compound treatment. Compounds that enhance the sensitivity of tumor cells to T-cell-mediated killing have comboscores >1. Among the top candidates that increased the sensitivity of treated tumor cells to T-cell killing were all three HSP90 inhibitors in the screen: 17-DMAG, BIIB021 and 17-AAG (Fig. 1a and Supplementary Fig. 2A), with 17-AAG being the compound with the highest combo score out of all 850 compounds. To validate these findings, we utilized a second generation HSP90 inhibitor, ganetespib, which has been reported to exhibit greater potency in preclinical tumor models and reduced ocular toxicity in rodents compared to 1st generation and other 2nd generation HSP90 inhibitors. Additionally, ganetespib also has a comparably better safety profile in patients^6, 7^. Confirming the screen results, varying concentrations of ganetespib increased the sensitivity of 2549 and 2338, and additional human melanoma cell lines 2400 and 2559 (*BRAF* V600E mutated), 2812 (wild type for *BRAF*, *NRAS* and *cKIT*) to T-cell-mediated killing (Fig. 1b and Supplementary Fig. 2B). Furthermore, the combination of ganetespib and TIL treatment was synergistic in tumor cell killing as indicated by combination indexes (CI) < 1 (Fig. 1c and Supplementary Fig. 2C) calculated by the Chou-Talalay method^8^. The combination treatment was synergistic in 2549 particularly at lower concentrations given that ganetespib treatment induced cleaved caspase 3 in 2549 at a higher percentage compared to the other cell lines (Supplementary Fig. 2D). Taken together, these data suggest HSP90 inhibitors as a combination partner to improve response to immunotherapy.

### HSP90 inhibitor effect on T-cell-mediated killing requires *IFIT* genes

**Fig. 2:**
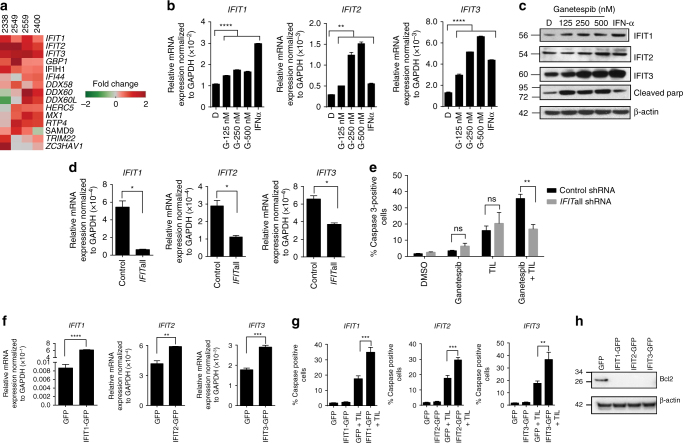
HSP90 inhibitor effect on T-cell-mediated killing requires *IFIT* genes. **a** Gene expression analysis of cell lines treated with ganetespib. **b**, **c** Quantitative real-time PCR (qRT-PCR) and western blot analysis showing upregulation of IFIT1, IFIT2 and IFIT3 following ganetespib treatment in human melanoma cell line 2400. D = DMSO and G = ganetespib. Interferon alpha (IFN-α) used as a positive control. An increase in cleaved PARP indicates efficacy of the HSP90 inhibition by ganetespib. **d** qRT-PCR to verify silencing of IFIT1, IFIT2 and IFIT3 in 2400. Control = cell line transduced with scrambled shRNA and IFITall = cell line transduced simultaneously with IFIT1, IFIT2 and IFIT3 shRNAs. **e** 2400 Control and IFITall cell lines treated with ganetespib at 250 nM, co-cultured with autologous T cells and assayed for cleaved caspase 3 by flow cytometry. **f** qRT-PCR verifying overexpression of IFIT1, IFIT2 and IFIT3 over GFP control in 2400. **g** 2400 GFP and IFIT overexpressing cell lines co-cultured with autologous T cells and assayed for cleaved caspase 3. **h** Western blots showing a decrease in BCL2 protein after overexpression of IFIT1, IFIT2 and IFIT3. The data represented as mean ± s.e.m. *P < 0.05; **P < 0.01; ***P < 0.001; ****P < 0.0001. Data are a representation of at least two independent studies

To mechanistically understand how HSP90 inhibition increased sensitivity of tumor cells to T-cell killing, we performed gene expression analysis of the human melanoma cell lines 2400, 2338, 2549 and 2559 treated with either DMSO, as a control, or ganetespib alone. Two commonly used bioinformatics tools, gene set enrichment analysis (GSEA) and Ingenuity Pathway Analysis (IPA), both implicated interferon response genes as being significantly upregulated following treatment with ganetespib, with interferon signaling being the highest-scoring canonical pathway by IPA (Supplementary Fig. 3A–C and Fig. 2a). Upregulation of interferon response genes in multiple melanoma cell lines by ganetespib was confirmed by quantitative real time PCR and Western blot analyses, most strongly for members of the IFN-induced protein with tetratricopeptide repeats (*IFIT*) gene family: *IFIT1, IFIT2* and *IFIT3* (Fig. 2b, c and Supplementary Fig. 4A, B). Furthermore, the *IFIT* genes were upregulated in vivo following treatment with ganetespib (Supplementary Fig. 3D). To determine the importance of the *IFIT* genes in the synergy observed between ganetespib treatment and T cells in tumor cell killing, *IFIT* gene expression was perturbed in tumor cells. *IFIT*-silenced and control tumor cell lines were generated by simultaneously transducing tumor cells with *IFIT1, IFIT2* and *IFIT3* small hairpin RNAs (shRNA) or with scrambled shRNA, respectively (Fig. 2d and Supplementary Fig. 4C). DMSO-treated control and *IFIT*-silenced cell lines were equally sensitive to T-cell killing. However, silencing the *IFIT* genes abrogated the enhanced killing of the melanoma cells potentiated by ganetespib (Fig. 2e and Supplementary Fig. 4D). Conversely, overexpressing *IFIT1, IFIT2* and *IFIT3* in human melanoma cell lines enhanced the sensitivity of human melanoma cell lines to T-cell killing, thereby recapitulating the effects of ganetespib treatment (Fig. 2f, g and Supplementary Fig. 4E, F). Interrogating apoptotic molecules upstream of caspase 3 revealed a dramatic decrease in BCL2 expression following overexpression of the *IFIT* genes (Fig. 2h and Supplementary Fig. 4G), suggesting that the *IFIT* genes promote sensitivity to apoptosis of the tumor cells. Taken together, our studies indicate that upregulation of *IFIT* genes is essential for the enhanced T-cell killing of melanoma cells following HSP90 inhibition.

### HSP90 inhibition potentiates immune checkpoint blockade therapy

**Fig. 3:**
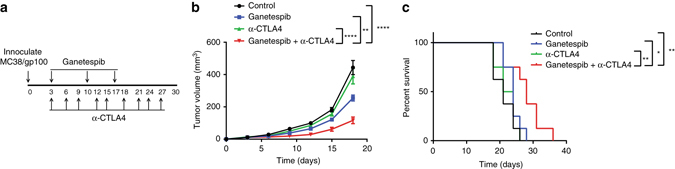
HSP90 inhibition potentiates responses to immune checkpoint blockade in vivo. **a** Treatment schedule: Treatment with ganetespib and anti-CTLA begun at the same time. Ganetespib was administered at 100 mg/kg per mouse once a week and anti-CTLA4 at 100 µg/mouse every 3 days. *n* = 8 mice. **b** Tumor volumes across treatment groups over time. **c** Survival of animals depicted by Kaplan–Meier curves. *n* = 8 mice. Vehicle = Solvent+Isotype antibody control. Mice were sacrificed when moribund or when tumor volume reached 1000 mm^3^ or tumors developed ulceration >3mm in diameter. The data represented as mean ± s.e.m. *P < 0.05; **P < 0.01; ***P < 0.001; ****P < 0.0001 by two way ANOVA. Data are a representation of at least two independent studies

We next assessed whether HSP90 inhibition could enhance responses to T-cell-directed immunotherapy in vivo. MC38/gp100 tumor-bearing mice were treated with solvent/antibody control (vehicle), ganetespib, anti-CTLA4 or anti-PD1, or the combination of ganetespib and anti-CTLA4 or anti-PD-1. Treatment with ganetespib was started at the same time as the antibody treatments (Fig. 3a). The combination of ganetespib and anti-CTLA4 conferred a better anti-tumor response, compared to either treatment alone (Fig. 3b) and improved survival compared to either treatment alone (Fig. 3c). The median survival for the combination of ganetespib and anti-CTLA4 were: vehicle = 21 days; ganetespib = 24 days; anti-CTLA4 = 22.5 days; and the combination of ganetespib and anti-CTLA4 = 28 days. The combination of ganetespib with anti-PD1 also conferred a better anti-tumor response and improved survival compared to either treatment alone (Supplementary Fig. 5A–C). The median survival for the combination of ganetespib and anti-PD1 were: vehicle = 21 days; ganetespib = 21 days; anti-PD1 = 22.5 days; and the combination of ganetespib and anti-PD1 = 28 days. Taken together, the results of the in vivo studies indicate that HSP90 inhibition enhances responses to immune checkpoint blockade therapy.

### Combining HSP90 inhibition and anti-CTLA4 enhances CD8 T-cell function

**Fig. 4:**
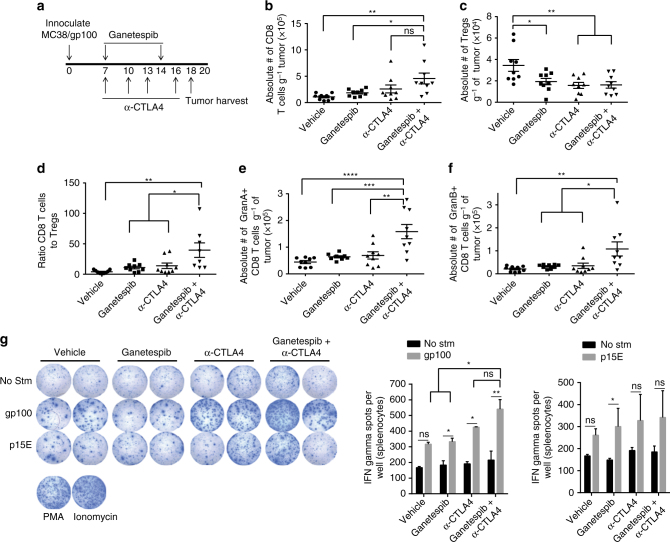
Combining HSP90 inhibition and anti-CTLA4 enhances CD8 T-cell function. **a** Treatment schedule: ganetespib was administered at 100 mg/kg per mouse once a week and anti-CTLA4 at 100 μg per mouse every 3 days. Two doses of ganetespib and 4 doses of anti-CTLA4 were administered. At day 18, mice were sacrificed, spleens harvested and processed, tumors were harvested and processed for RNA and immune cell infiltration profiled via flow cytometry. **b** Absolute number of CD8 T cells infiltrating the tumor normalized to tumor weight. **c** Absolute number of T regulatory cells infiltrating tumor normalized to tumor weight. **d** Ratio of CD8 T cells to Tregs infiltrating the tumor. **e** Absolute number of granzyme A positive CD8 T cells normalized to tumor weight. **f** Absolute number of granzyme B positive CD8 T cells normalized to tumor weight. *n* = 9 mice. **g** ELISPOTS showing gp100 and P15E-specific cells from splenocytes of treated mice. The data represented as mean ± s.e.m. *P < 0.05; **P < 0.01; ***P < 0.001; ****P < 0.0001 by one way ANOVA for B-F or paired two tailed Student’s *t*-test for G. Data are a representation of at least two independent studies

To investigate whether HSP90 inhibition and immune checkpoint blockade treatments modulate the immune cell population infiltrating the tumor, C57BL/6 mice were treated with vehicle, ganetespib, anti-CTLA4, or the combination of ganetespib and anti-CTLA4, starting day 7 after inoculation with MC38/gp100 tumors (Fig. 4a). In tumors harvested on day 18 after tumor inoculation, the absolute number of CD8 T cells (Fig. 4b) and Ki67-positive CD8 T cells (Supplementary Fig. 6A) were highest in the combination treatment, but the differences between the anti-CTLA4 only and combination groups were not statistically significant, suggesting that ganetespib treatment does not further increase number or proliferation of CD8 T cells in the tumor. However, the number of T regulatory cells (Tregs) was significantly decreased in the treatment groups compared to the vehicle (Fig. 4c). Other studies have shown that anti-CTLA4 and HSP90 inhibitor treatments alone have been shown to decrease the number of tumor-infiltrating Tregs^9, 10^. The decrease in the number of Tregs resulted in an increased CD8 T cell to Treg ratio in the treatment groups, with the differences between the combination groups and the other groups being statistically significant (Fig. 4d). Significant increases in the expression of the trafficking chemokines, CXCL9 and CXCL10, were observed with the combination treatment of ganetespib and anti-CTLA4, relative to vehicle control. In addition, a decrease in the immunosuppressive cytokine VEGFA was observed in the treatment groups including ganetespib (Supplementary Fig. 6E), but the effect did not appear to be significantly enhanced by the inclusion of anti-CTLA4. Other cell populations such as myeloid-derived suppressor cells (MDSCs) and effector CD4 T cells remained unchanged (Supplementary Fig. 6B, C).

To investigate whether the increase in CD8 T cell to Treg ratio had an impact on CD8 T-cell effector function, production of the cytotoxic molecules granzyme A and granzyme B by CD8 T cells was assessed. The number of CD8 T cells making granzymes A and B was significantly higher in the combination treatment group compared to the other treatment groups (Figs. 4e, f, Supplementary Fig. 6D), suggesting that the combination treatment enhanced the effector function of CD8 T cells infiltrating the tumor.

Furthermore, to interrogate whether the generation of antigen-specific cells was affected by treatment, splenocytes isolated from mice treated with ganetespib, with and without anti-CTLA4, were stimulated ex vivo with either DMSO (control), gp100, or p15E peptides (MHC I restricted peptides expressed by the MC38/gp100 tumor), and IFN-gamma production was assessed by ELISPOT. Results indicated a significant increase in IFN-gamma producing cells following stimulation with gp100 peptide in the treatments groups, with the increase being the most dramatic in the combination treatment group (Fig. 4g), suggesting that the treatment of ganetespib and anti-CTLA4 enhances antigen-specific CD8 T-cell generation. Stimulation with P15E peptide showed a similar trend although the increase was not statistically significant in the anti-CTLA4 and combination groups. Taken together, these results suggest that the combination of HSP90 inhibition and anti-CTLA4 therapy enhances the generation of antigen-specific CD8 T cells and the effector function of CD8 T cells.

## Discussion

In this study, we demonstrated HSP90 inhibition as a promising combination with immunotherapy. HSP90 is important in maintaining the structural integrity of over 200 client proteins, thereby regulating a variety of cellular processes. Many client proteins of HSP90 are known oncogenic drivers that can regulate tumor intrinsic pathways, some of which may provide a route of interference with response to immunotherapy^11–14^. Although inhibition of HSP90 has received attention for therapeutic purposes in solid tumors and hematologic malignancies, HSP90 inhibition has shown limited responses as single agents in cancer patients^15, 16^. Studies from our group and others provide evidence for combining HSP90 inhibition with immunotherapy. Preclinical studies from the Storkus group using the first generation HSP90 inhibitor 17-DMAG, provided initial evidence of HSP90 as a tumor intrinsic molecule that could be targeted to enhance responses to immunotherapy in a vaccine model^17, 18^. We now provide evidence that HSP90 inhibition, using the potent 2nd generation inhibitor ganetespib, enhances T cell-mediated killing of melanoma cells. We also show, for the first time, that ganetespib potentiates responses to anti-CTLA4 and anti-PD-1 immunotherapy in vivo.

While HSP90 inhibitors have been exploited for their cytotoxic effects on tumor cells, the role of HSP90 in immunomodulation, as described in in ex vivo and in vivo studies^19–21^, raises the possibility that prolonged or continuous exposure to HSP90 inhibitor treatments might be detrimental to an anti-tumor immune response. In our in vivo studies we did not observe impairment of antigen-specific CD8 T cells or total CD8 T-cell generation, function and proliferation in the setting of ganetespib treatment. The only cell population decreased within the tumor microenvironment following treatment with ganetespib were Tregs. This is consistent with a recent study depicting the essential role of the HSP90 paralog, GP96, in maintaining FOXP3 expression and production of TGF-β by Tregs. As such, mice with a homozygous deletion of GP96 had significantly decreased amounts of Tregs^22^. This decrease in Tregs observed with our treatments contributed to the increased CD8 T cell to Treg ratios. In addition, interrogation of several chemokines and cytokines known to be modulated in our tumor model^23^ revealed an increase in trafficking chemokines CXCL9 and CXCL10, and a decrease in the immunosuppressive cytokine VEGFA, consistent with studies showing these changes enhance CD8 T-cell function^5, 24–26^. Our treatment schedule calls for administration of ganetespib, at 100 mg/kg once a week for 3 weeks, concurrently with immune checkpoint blockade therapy, compared to a regimen of 50 mg/kg of ganetespib alone twice a week for 14 weeks in a murine model of systemic lupus erythematosus or a schedule of once daily for 14 weeks in an epidermolysis bullosa acquisita model^19, 27^. This intermittent administration of ganetespib in our regimen may avoid potential immune suppression, while maintaining therapeutic benefit. Furthermore, given the selective retention and sustained chaperone activity inhibition of ganetespib within the tumors, which express up to 10-fold more HSP90 complexes, compared to normal tissue, intermittent dosing regimens may lead to effective and sustained inhibition of chaperone inhibitory activity within the tumor compartment, while restricting systemic immune system drug exposures^28, 29^. Therefore, the dose and dose regimen for of HSP90 inhibitor treatments for oncology indications should be carefully considered, on a case-by-case basis.

We also demonstrate for the first time that HSP90 inhibition upregulates interferon response genes in the tumor, notably *IFIT1*, *IFIT2* and *IFIT3*, both in vitro and in vivo. Indeed, recent studies reveal that a higher expression of type I interferon response genes, including the genes interrogated in this study, have been associated with long term benefits to anti-CTLA4 immunotherapy across multiple cancer types^30^. The *IFIT* genes whose major roles have been previously described in response to viral infections have also shown a role in tumor biology. Particularly, *IFIT2* overexpression in tumor cells promotes tumor cell death^31^. We now show that *IFIT2* and other family members *IFIT1* and *IFIT3*, when overexpressed in tumor cells, enhance T-cell killing of tumor cells. Importantly, silencing of these genes abrogated the enhanced T-cell killing of melanoma cells following HSP90 inhibition. This provides additional evidence that these genes play an essential role in inducing death of tumor cells and their importance in potentiating response to T-cell-mediated immunotherapy. Moreover, with the recent identification of aberrations in the IFN pathway as a mechanism of immune resistance^32–34^, this provides another avenue in investigations of whether HSP90 inhibitors may play a role in reversing this resistance.

Taken together, our study provides evidence that HSP90 inhibition can potentiate T-cell-mediated anti-tumor immune responses and supports exploration of the combination of immunotherapy and HSP90 inhibitors in the clinic.

## Methods

### Study approval

Six to 12-week-old female C57BL/6 mice were purchased from the Charles River Frederick research model facility (Bethesda, MD). Mice were handled in accordance with protocols approved by the Institutional Animal Care and Use Committee. The MC38/gp100 cell line was established as previously described^23^. Human melanoma cell lines 2338, 2400, 2549, 2559, 2812 and their autologous tumor infiltrating lymphocytes (TILs) cells were established from metastatic lesions isolated during palliative surgery at MD Anderson Cancer Center using an Institutional Review Board (IRB) approved laboratory protocol (LAB06-0755)^35^. All cell lines were maintained in complete cell culture medium as previously described^5^. All cell lines were routinely tested for mycoplasma and were mycoplasma negative. All cell lines were verified by short tandem repeat fingerprinting or matching mutational profiles. All in vitro experiments were performed in duplicate or triplicate and in vivo experiments utilized 8–10 mice based on established protocols or previous experience with model systems.

### Compound screen

A library of 850 bioactive compounds was curated by Florian Mueller and Nikunj Satani (MDACC) and purchased from Selleckchem. A total of 50,000 patient derived human melanoma cells were labelled with the cell tracker dye, DDAO. The labelled cells were then treated with either 1 µM of each compound or DMSO alone for 24 h at 37 °C in triplicate in a 96-well format or treated first with the compound or DMSO, washed off and co-cultured with autologous T cells at a predetermined ratio for 3 h. The cells were then assayed for cleaved caspase 3 via flow cytometry as previously described^36^. A comboscore was calculated based on observed changes in the percentage of T-cell-induced apoptosis in tumor cells with or without compound treatment. Compounds that enhance the sensitivity of tumor cells to T-cell-mediated killing have comboscores >1. Comboscore = ((% Caspase by Compound & TILs−% caspase by Compound)/(% caspase by TILs))^2^.

### Gene expression profiling (GEP) and analysis

The patient-derived human melanoma cell lines 2338, 2400, 2549 and 2559 were cultured in growth medium (RPMI supplemented with 10% FCS and 1% Normocin) and plated at no more than 75% confluence for treatment with ganetespib. Cells were treated in duplicate with DMSO as a control or 125 nM of ganetespib for 24 h at 37 °C. After drug treatment, cells were washed with sterile PBS and then trypsinized. Collected cells were then washed twice with sterile PBS and spun. Supernatant was aspirated, and cell pellets flash frozen in liquid nitrogen. Frozen cell pellets were shipped to Expression Analysis (Durham, NC) for RNA isolation and analysis using Illumina human HT12v4.0 arrays. For each cell line and gene probe, the log2 values of ganetespib duplicates were averaged and subtracted by the log2 values of DMSO samples. The average of these subtracted values, across all four lines, was then used to create a gene rank for analysis by gene set enrichment analysis (GSEA) software, which uses a Kolgorimov-Smirnov statistic to determine the significance of distribution of a set of genes within a larger, ranked data set^37^. GEP data were also analyzed using the comparison analysis tool in Ingenuity Pathway Analysis software, which scores canonical pathways by their consistent change across all samples, with a positive *z* score indicating an increase in expression or activation of the canonical pathway, and a negative *z* score indicating a decrease.

### Quantitative real time PCR

Total RNA was extracted using RNAeasy plus mini kit (QIAGEN). 2 μg of RNA was reverse transcribed to cDNA using the High Capacity RNA-to-cDNA kit (Thermofisher). Quantitative real time PCR was performed, in triplicate, using the Taqman gene expression assay system according to manufacturer’s instructions (Life Technologies). A list of the gene expression assays used is provided in the Supplementary Table 1. Samples were normalized to GAPDH expression level using 2^−∆∆CT^ method.

### Western blots

Samples were lysed in RIPA lysis buffer system (Santa Cruz) according to manufacturer’s instructions. 50 µg of protein in cell lysates were separated in 4–20% SDS polyacrylamide gels and transferred to nitrocellulose membranes. Membranes were incubated with the following primary antibodies against IFIT1 (Cell Signaling), IFIT2 (Abcam), IFIT3 (Abcam), cleaved PARP (Cell signaling), BCL2 (Cell signaling) and β-actin (Cell Signaling). Anti-rabbit and anti-mouse IgG antibodies tagged with horseradish peroxidase (Cell signaling) were used. Uncropped scans of the blots are available in Supplementary Figs. 7 and 8.

### Generation of ORF-expressing tumor cells for in vitro T-cell-killing assay

Viral particles containing ORF gene-of-interest were used to infect patient-derived melanoma cells. To test the effect of overexpression of *IFIT* genes, freshly transduced cells were co-cultured, in triplicate, with autologous TILs for 3 h and assayed for cleaved caspase 3. ORF-positive cells were gated based on the expression of tagged GFP via flow cytometry analysis.

### Generation of shRNA-expressing tumor cells lines

Viral particles containing either control shRNA or shRNAs of gene-of-interest were used to infect patient derived melanoma cells. Stable cell lines expressing shRNAs targeting the *IFIT* genes were generated by 2-week puromycin treatment after viral transduction. These established stable cell lines were used for in vitro experiments. To test the effect of the knockdown of the *IFIT* genes to the synergistic effect of ganetespib and T-cell killing, freshly transduced cells were treated, in triplicate, with either ganestespib or DMSO for 24 h and assayed as described above.

### Treatments, immune cell tumor infiltration and ELIPOT analyses

A sample size of 8–10 mice was used per experiment to ensure adequate power. Investigator handling mice was blinded to the groups. The HSP90 inhibitor ganetespib was provided by Synta Pharmaceuticals (Lexington, MA). MC38/gp100 tumor-bearing mice were randomized into treatment groups and treated with a dose of 100 mg/kg once weekly. Anti-CTLA-4 (9H10) was purchased from BioXcell and was administered every 3 days at a dose of 100 µg/mouse. Isotype antibody control used was Polyclonal Armenian Hamster IgG. Anti-PD-1 (clone: 29 F.1A12, 135204, BioLegend) was administered every 3 days at a dose of 200 µg/mouse. Isotype control was Rat IgG2A (clone: RTK2758, 400543, BioLegend). Treated mice were sacrificed on day 18 following tumor inoculation and tumors and spleens were harvested. Tumors were dissected into fragments by cutting, digested in tumor digestion buffer for 1 h at 37 °C and filtered through 45 µm nylon mesh. The tumor digestion buffer was made by dissolving 1 mg/ml collagenase, 100 µg/ml hyaluronidase and 20 mg/ml Dnase (Sigma-Aldrich, St. Louis, MO) in RPMI medium. Cell suspensions were stained for intracellular and extracellular protein markers of interest. Stained samples were acquired on a BD LSRFORTESSA X-20 instrument and data analyzed using Flowjo software. Staining antibodies were as follows: Anti-CD45 (30-F11, Tonbo Biosciences), anti-CD8 (53-6.7, Tonbo Biosciences), anti-CD3 (145-2C11, Tonbo Bioscience), anti-CD4 (RMA-5, Tonbo Biosciences), anti-Foxp3 (FJK-16s, eBioscience), anti-CD25 (PC61.5, eBioscience), anti-Gr1 (RB6-8C5, Tonbo Biosciences), anti-Ly6C (HK1.4, Biolegend), anti-CD11b (M1/70, Tonbo Biosciences), anti CD11c (N418, Tonbo Biosciences), anti-F4/80 (BM8.1, Tonbo Biosciences), anti-granzyme B (GB11, BD Bioscience), anti-granzyme A (GzA-3G8.5, Affymetrix Inc.), anti-Ki67 (SolA15, Affymetrix Inc.) and anti-cleaved caspase 3 (550821, BD Bioscience). Single cell suspensions of splenocytes were prepared and erythrocytes depleted using ACK lysis buffer (Life technologies). The murine IFN-gamma single-color enzymatic ELISPOT assay kit from Immunospot was used to assess IFN-gamma producing cells. Splenocytes from 3 mice with similar tumor weights were combined within each treatment group and 300,000 splenocytes were incubated with either DMSO, gp100 peptide (KVPRNQDWL) at 1 μg/ml or p15E peptide (KSPWFTTL) at 1 μg/ml for 24 h at 37 °C and assayed according to the manufacturer’s instructions. Spots were counted and quantified using the CTL Immunospot reader.

### Statistical analyses

The data are represented as mean ± s.e.m. Comparisons of differences in continuous variables between 2 groups were done using unpaired Student *t*-tests. Comparisons of differences in continuous variables within a group (DMSO vs peptide stimulation) were done using paired Student *t*-tests. Differences in tumor size and T-cell numbers among different treatments were evaluated by ANOVA repeated-measures function. The Kaplan–Meier method and log-rank test were used to compare survival between groups. Variances were similar between groups statistically compared. *P* values are based on two-tailed tests, with *P* < 0.05 considered statistically significant. Graphs were generated using GraphPad Prism 6 and Tableau software. Statistical analyses were performed using GraphPad Prism 6.

### Data availability

Microarray data supporting the findings of this study have been deposited in GEO repository with the accession code GSE100317 (https://www.ncbi.nlm.nih.gov/geo/query/acc.cgi?acc=GSE100317).

## Electronic supplementary material

Supplementary Information
