# Supplementary material for: HSP90 inhibition enhances cancer immunotherapy by upregulating interferon response genes
Source: Nat Commun. 2017 Sep 6;8:451. doi: 10.1038/s41467-017-00449-z (PMC5587668; doi:10.1038/s41467-017-00449-z)
Supplement: Supplementary file 1 — Supplementary Information [file 41467_2017_449_MOESM1_ESM.pdf]

Description of Supplementary Files

File Name: Supplementary Information
Description: Supplementary Figures and Supplementary Table

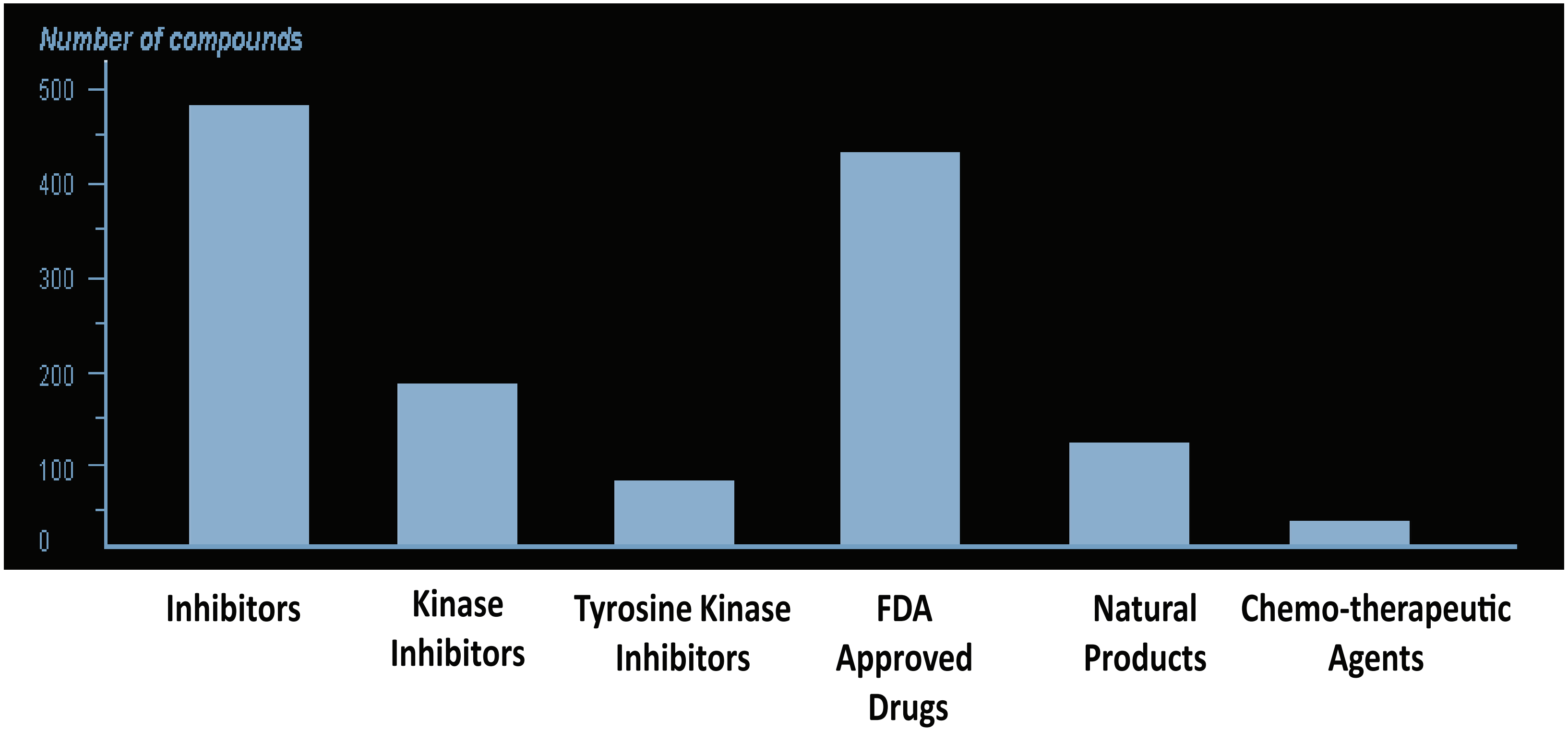

Supplementary Figure 1: 850 bioactive compound classification from selleckchem.

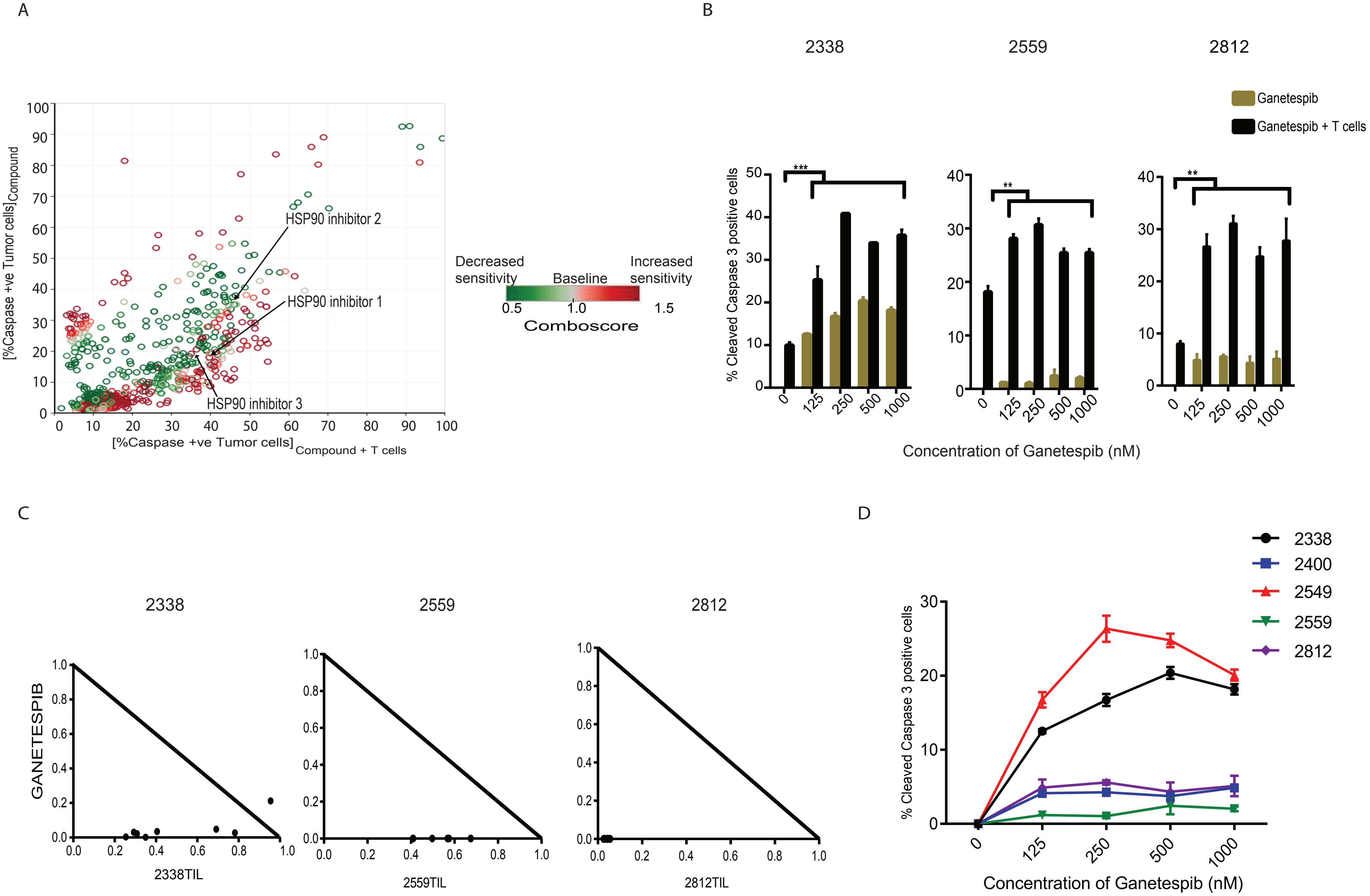

Supplementary Figure 2: HSP90 inhibition enhances T cell mediated killing of melanoma cells. (A) Tableau depiction of screen results from patient derived cell line 2338. HSP90 inhibitors 1 - 17-DMAG, 2 – BIIB021 and 3 -17-AAG are highlighted. (B) Cleaved caspase 3 percentage following treatment with varying concentrations of ganetespib and autologous TILs in human melanoma cell lines 2338, 2559 and 2812. (C) Normalized isobolograms depicting the synergism between ganetespib and T cell killing. Combination indexes < 1 indicate synergy. (D) Caspase 3 cleavage induced by ganetespib alone across all five cell lines. The data represented as mean ± SEM. **P < 0.01; ***P < 0.001 by unpaired two tialed student T test. Data are a representation of at least two independent studies.

A

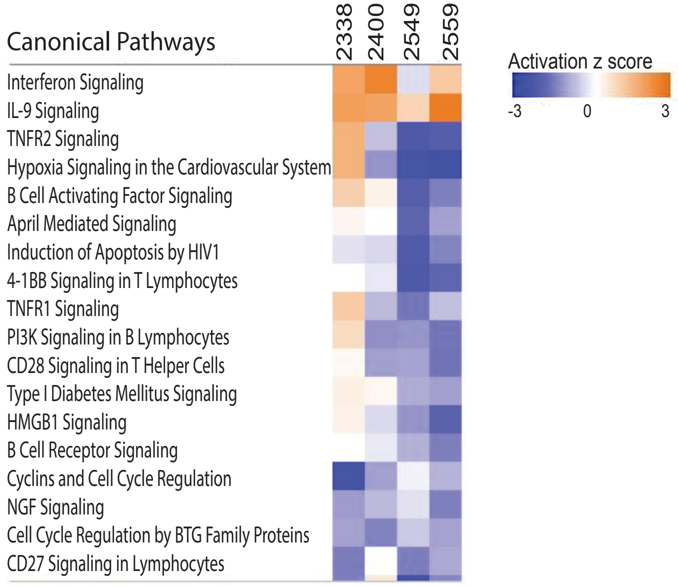

B

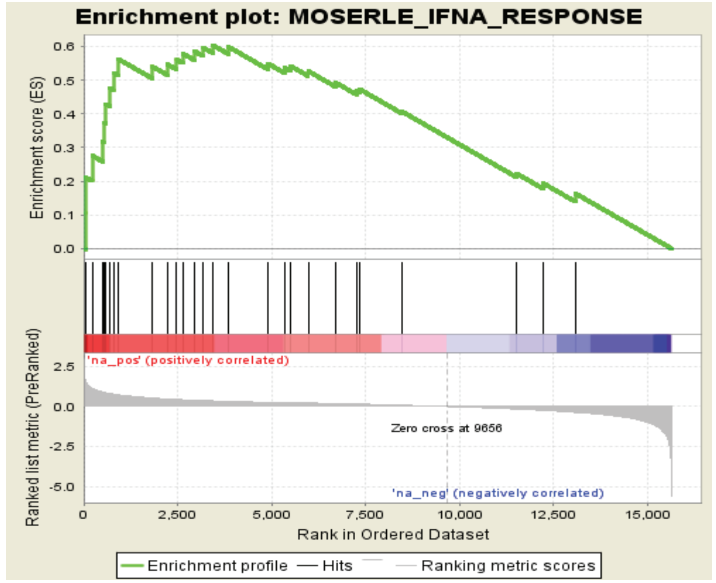

C

NAME	PROBE	RANK IN GENE LIST	RANK METRIC SCORE	RUNNING ES	CORE ENRICHMENT
row_0	IFIT3	44	1.60714376	0.10464722	Yes
row_1	IFIT2	45	1.604997277	0.21196944	Yes
row_2	EPSTI1	223	1.127469301	0.27602237	Yes
row_3	GBP1	512	0.895565569	0.31745812	Yes
row_4	RTP4	548	0.880248189	0.37407613	Yes
row_5	IFIT1	586	0.860438883	0.42924145	Yes
row_6	DDX60	705	0.807804108	0.47569853	Yes
row_7	MX1	788	0.779251099	0.52255243	Yes
row_8	IFIH1	921	0.734060228	0.56318164	Yes
row_9	ZC3HAV1	1834	0.53478986	0.5405214	Yes
row_10	HERC5	2221	0.475829691	0.5476128	Yes
row_11	DDX58	2476	0.445198894	0.56111157	Yes
row_12	SAMD9	2639	0.427447706	0.5793167	Yes
row_13	TRIM22	2938	0.400018007	0.58697575	Yes
row_14	DDX60L	3164	0.379113734	0.59791327	Yes
row_15	IFI44	3441	0.356272995	0.60405654	Yes
row_16	OAS2	3849	0.327158958	0.59986144	No
row_17	TNFSF10	4901	0.260549664	0.5499595	No
row_18	USP18	5340	0.236566901	0.537721	No
row_19	CXCL10	5504	0.229704812	0.54263943	No
row_20	RSAD2	5973	0.219200432	0.52731794	No
row_21	SAMD9L	6695	0.188416034	0.4937315	No
row_22	IFIT5	7257	0.160109058	0.4685014	No
row_23	IFI16	7352	0.155639321	0.47288722	No
row_24	IFI44L	8455	0.087456793	0.408144	No
row_25	OAS1	11513	-0.164081007	0.22329223	No
row_26	STAT1	12228	-0.242892444	0.19379687	No
row_27	IFITM1	13095	-0.367514521	0.16289796	No

D

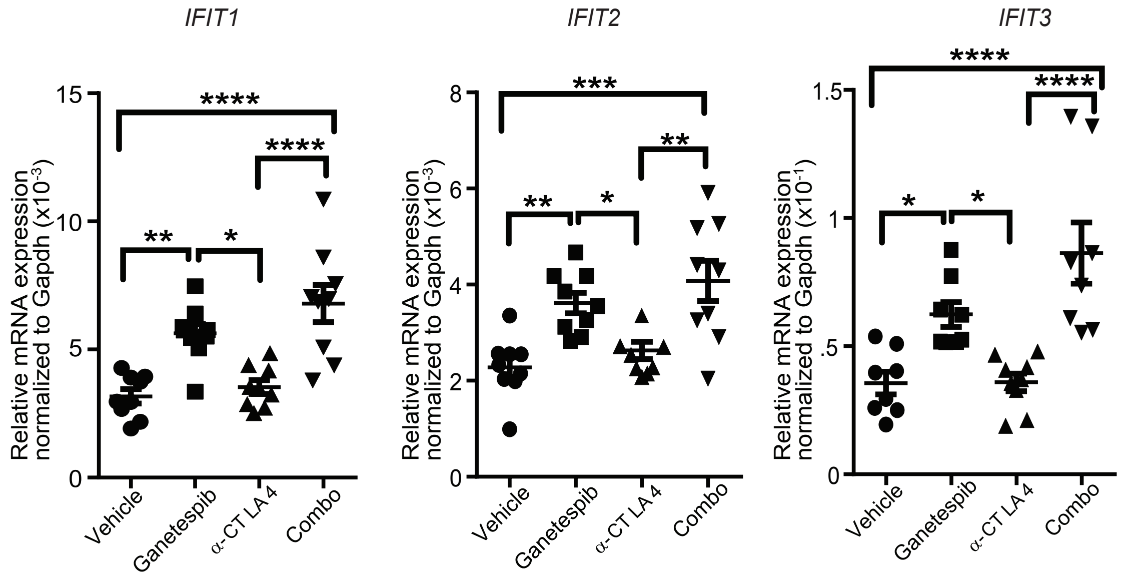

Supplementary Figure 3: Interferon response genes are upregulated following HSP90 inhibition. (A) Hierarchical clustering of canonical pathways across samples using comparison analysis in the Ingenuity pathway analysis software. Heat map shows interferon signaling the most consistently upregulated pathway across all four cell lines. (B)&(C) GSEA shows interferon response genes upregulated following treatment with ganetespib. (D) Graphs depict the in vivo expression of IFIT genes by qRT-PCR. Combo = ganetespib + α-CTLA4. The data represented as mean ± SEM. *P < 0.05; **P < 0.01; ***P < 0.001; ****P < 0.0001 by one way anova n = 9 mice.

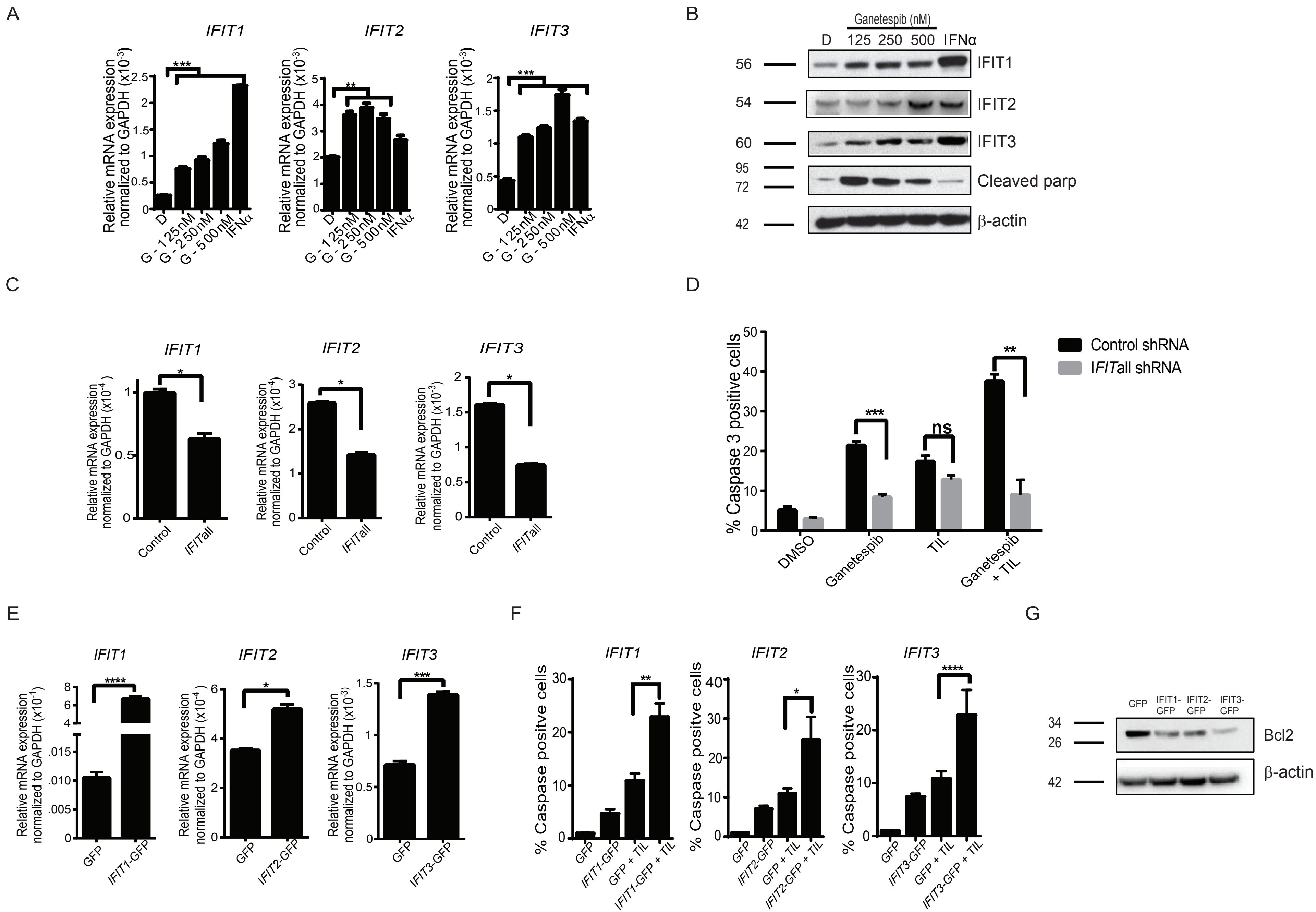

Supplementary Figure 4: HSP90 inhibitor effect on T cell mediated killing requires IFIT genes. (A)&(B) Quantitative real time PCR (qRT-PCR) and western blot analysis showing upregulation of IFIT1, IFIT2 and IFIT3 following ganetespib treatment in human melanoma cell line 2549. D = DMSO and G = Ganetespib. Interferon alpha (IFN-α) used as a positive control. An increase in cleaved PARP indicates efficacy of the HSP90 inhibition by ganetespib. (C) qRT-PCR to verify silencing of IFIT1, IFIT2 and IFIT3 in 2549. Control = 2549 transduced with scrambled shRNA and IFITall = 2549 transduced with IFIT1, IFIT2 and IFIT3 shRNAs simultaneously. (D) 2549 Control and IFITall cell lines treated with ganetespib at 250nM, co-cultured with autologous T cells and assayed for cleaved caspase 3. (E) qRT-PCR verifying overexpression of IFIT1, IFIT2 and IFIT3 over GFP control in 2549. (F) 2549 GFP and IFIT overexpressing cell lines co-cultured with autologous T cells and assayed for cleaved caspase 3. (G) Western blots showing a decrease in BCL2 protein after overexpression of IFIT1, IFIT2 and IFIT3. The data represented as mean ± SEM. *$P < 0.05$; **$P < 0.01$; ***$P < 0.001$; ****$P < 0.0001$ by unpaired two tailed student T test. Data are a representation of at least two independent studies.

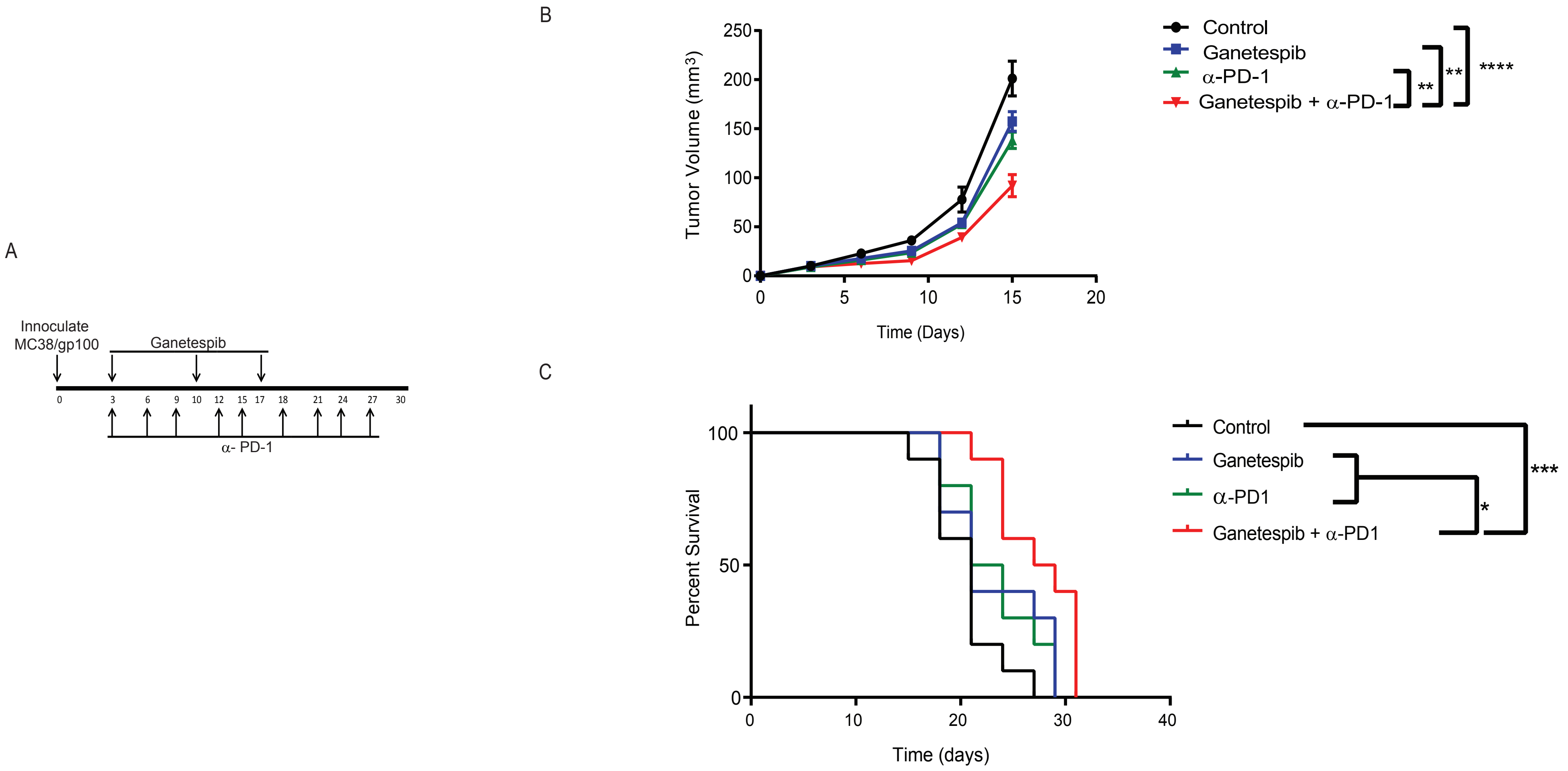

Supplementary Figure 5: HSP90 inhibition potentiates responses to immune checkpoint blockade in vivo. (A) Treatment schedule: Treatment with ganetespib and anti-PD1 begun at the same time. Ganetespib was administered at 100mg/kg per mouse once a week and anti-PD1 at 200ug per mouse every 3 days. n = 8 mice (B) Tumor volumes across treatment groups over time. (C) Survival of animals depicted by Kaplan-Meier curves. n = 8 mice. Vehicle = Solvent + Isotype antibody control. Mice were sacrificed when moribund or when tumor volume reached 1000mm3 or tumors developed ulceration >3mm in diameter. The data represented as mean ± SEM. *P < 0.05; **P < 0.01; ***P < 0.001; ****P < 0.0001 by two way anova. Data are a representation of at least two independent studies.

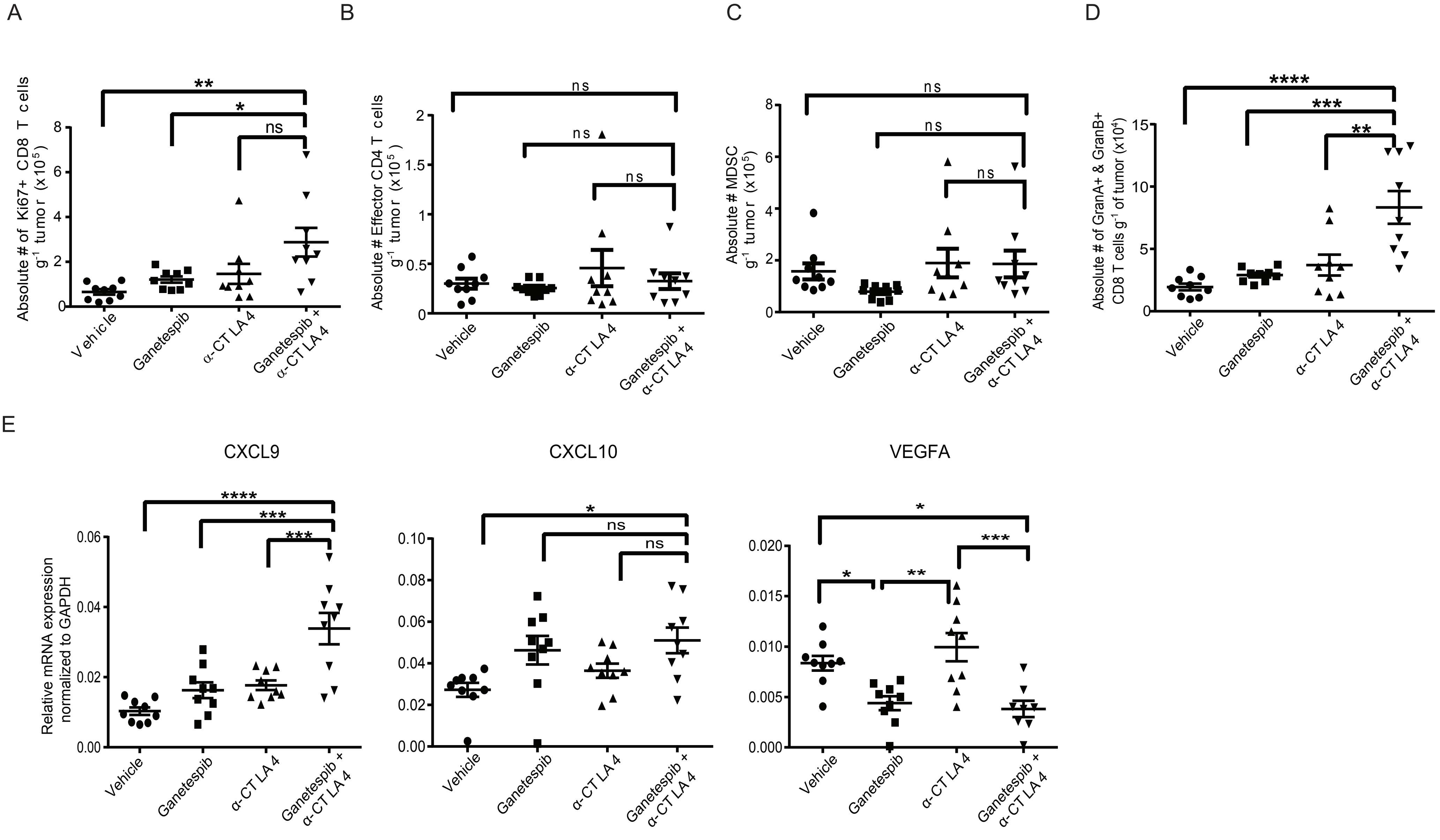

Supplementary Figure 6: The combination of HSP90 inhibition and anti-CTLA4 treatment modulates the immune cell population in the tumor. (A) Absolute number of Ki67 positive CD8 T cells infiltrating the tumor normalized to tumor weight. (B) Absolute number of effector CD4 T cells (FOXP3 negative) infiltrating tumor normalized to tumor weight (C) Absolute number of MDSCs infiltrating tumor normalized to tumor weight. (D) Absolute number of granzymes A and B double positive CD8 T cells infiltrating the tumor normalized to tumor weight. (E) mRNA expression of chemokines and cytokines in tumor cells following treatment with ganetespib and anti-CTLA4. The data represented as mean ± SEM. $*P < 0.05$; $**P < 0.01$; $***P < 0.001$; $****P < 0.0001$ by one way anova. n = 9 mice. Data are a representation of at least two independent experiments.

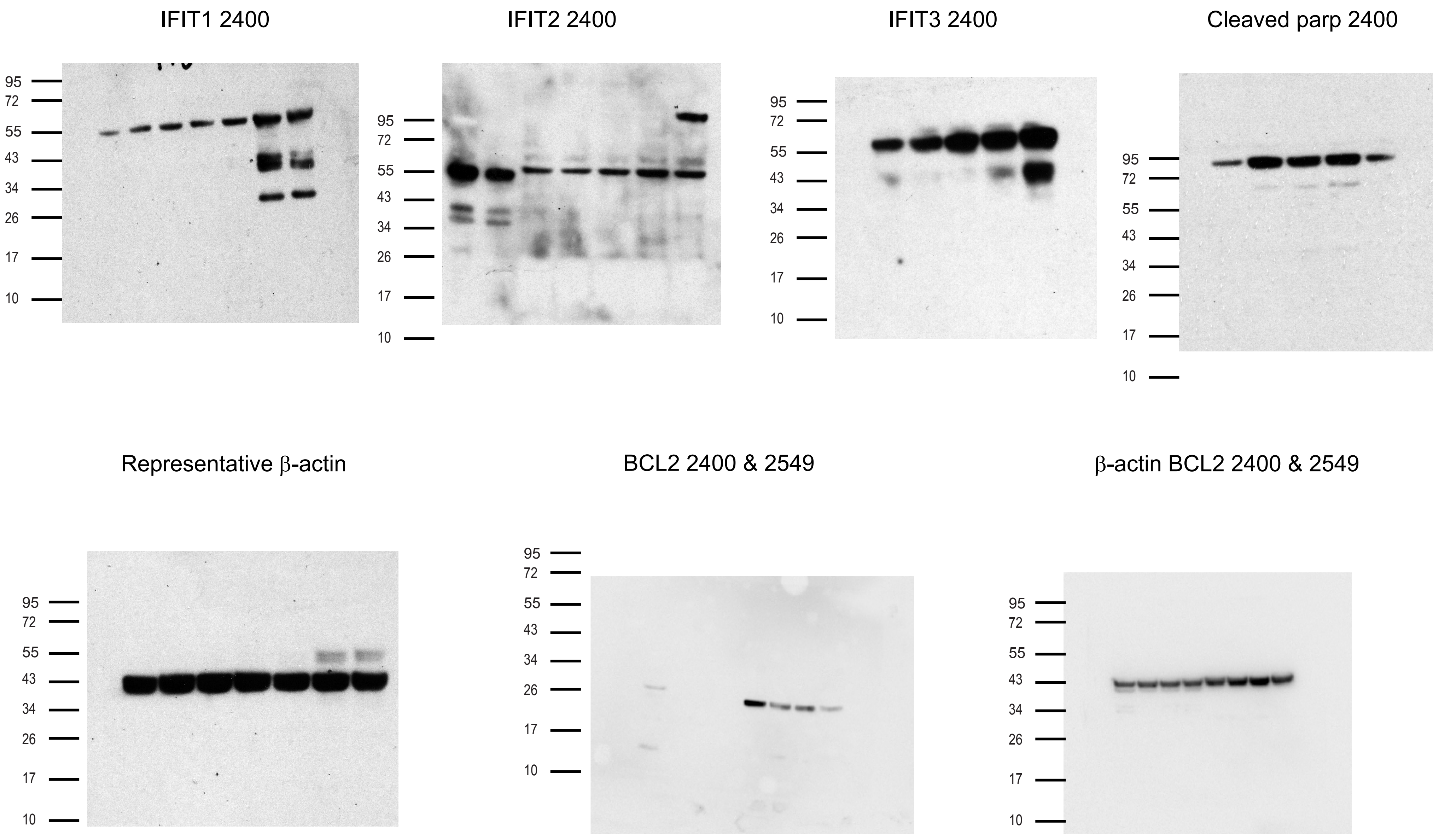

Supplementary Figure 7: Uncut western blots for cell lines 2400 and 2549.

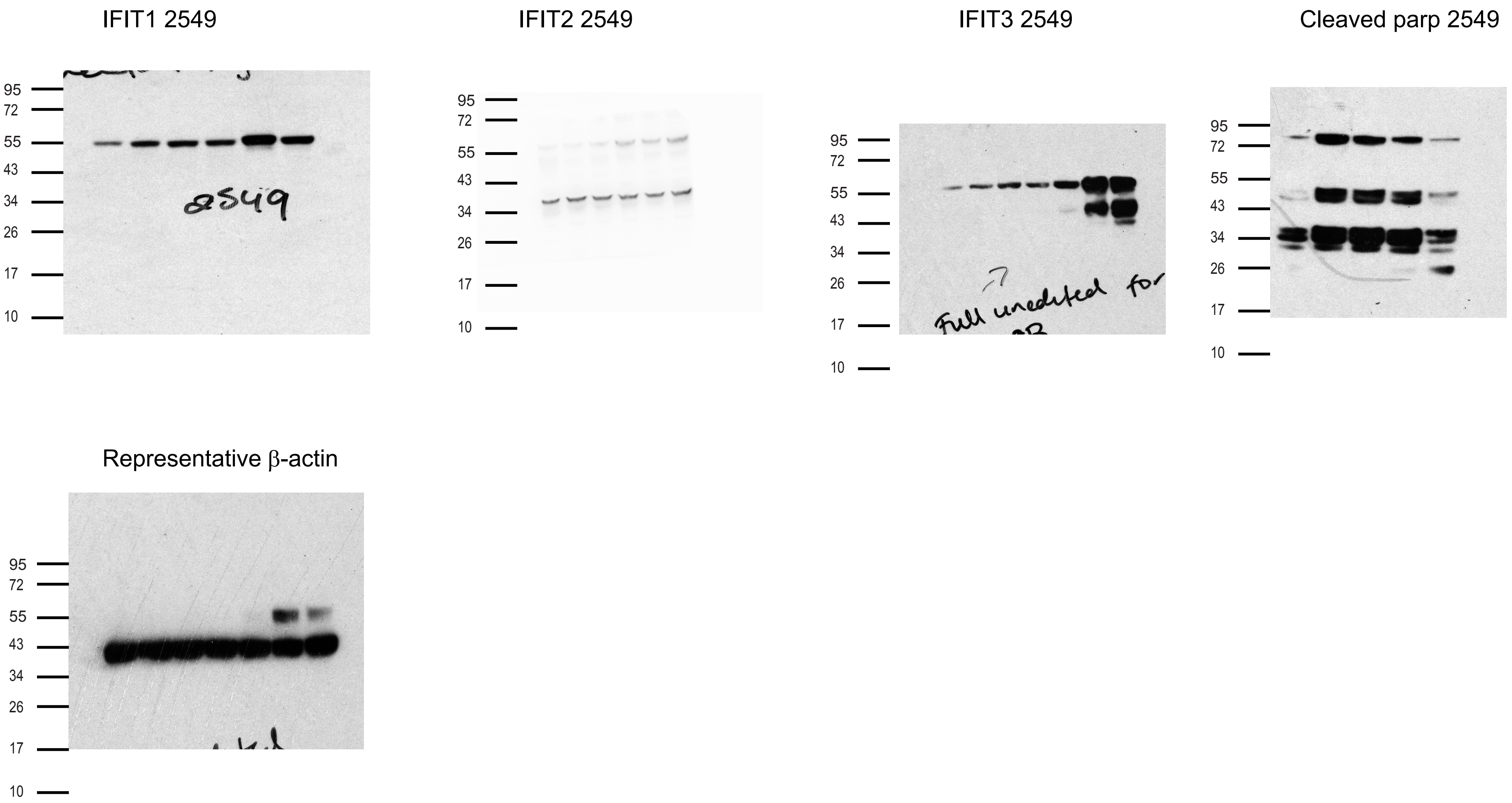

Supplementary Figure 8: Uncut western blots for cell line 2549.

Gene symbol	Species	Assay ID
GAPDH	Human	Hs02758991_g1
IFIT1	Human	Hs03027069_s1
IFIT2	Human	Hs01922738_s1
IFIT3	Human	Hs01922752_s1
Gapdh	Mouse	Mm03302249_g1
Ifit1	Mouse	Mm00515153_m1
Ifit2	Mouse	Mm00492606_m1
Ifit3	Mouse	Mm01704846_s1
Cxcl9	Mouse	Mm00434946_m1
Cxcl10	Mouse	Mm00445235_m1
Vegfa	Mouse	Mm00437306_m1

Supplementary Table 1: Taqman Real time PCR primers assay IDs
